# *In Situ* Microbial Community Succession on Mild Steel in Estuarine and Marine Environments: Exploring the Role of Iron-Oxidizing Bacteria

**DOI:** 10.3389/fmicb.2016.00767

**Published:** 2016-05-24

**Authors:** Joyce M. McBeth, David Emerson

**Affiliations:** ^1^Bigelow Laboratory for Ocean SciencesEast Boothbay, ME USA; ^2^Department of Geological Sciences, University of SaskatchewanSaskatoon, SK, Canada

**Keywords:** succession, Epsilonproteobacteria, Zetaproteobacteria, sulfate-reducing bacteria, MIC

## Abstract

Microbiologically influenced corrosion (MIC) is a complex biogeochemical process involving interactions between microbes, metals, minerals, and their environment. We hypothesized that sediment-derived iron-oxidizing bacteria (FeOB) would colonize and become numerically abundant on steel surfaces incubated in coastal marine environments. To test this, steel coupons were incubated on sediments over 40 days, and samples were taken at regular intervals to examine microbial community succession. The experiments were conducted at two locations: (1) a brackish salt marsh stream and (2) a coastal marine bay. We analyzed DNA extracted from the MIC biofilms for bacterial diversity using high-throughput amplicon sequencing of the SSU rRNA gene, and two coupons from the coastal site were single cell sorted and screened for the SSU rRNA gene. We quantified communities of Zetaproteobacteria, sulfate-reducing bacteria (SRB), and total bacteria and archaea using qPCR analyses. Zetaproteobacteria and SRB were identified in the sequencing data and qPCR analyses for samples collected throughout the incubations and were also present in adjacent sediments. At the brackish site, the diversity of Zetaproteobacteria was lower on the steel compared to sediments, consistent with the expected enrichment of FeOB on steel. Their numbers increased rapidly over the first 10 days. At the marine site, Zetaproteobacteria and other known FeOB were not detected in sediments; however, the numbers of Zetaproteobacteria increased dramatically within 10 days on the steel surface, although their diversity was nearly clonal. Iron oxyhydroxide stalk biosignatures were observed on the steel and in earlier enrichment culture studies; this is evidence that the Zetaproteobacteria identified in the qPCR, pyrosequencing, and single cell data were likely FeOB. In the brackish environment, members of freshwater FeOB were also present, but were absent in the fully marine site. This work indicates there is a successional pattern in the colonization of steel surfaces with FeOB being early colonizers; over time the MIC community matures to include other members that may help accelerate corrosion. This work also shows there is a reservoir for Zetaproteobacteria in coastal sediment habitats, where they may influence the coastal iron cycle, and can rapidly colonize steel surfaces or other sources of Fe(II) when available.

## Introduction

It is generally believed that the costs associated with corrosion are equivalent to about 3% of a nation’s gross domestic product; corrosion of steel costs US taxpayers billions of dollars a year in damage to infrastructure (NACE International, 2002). In the marine environment, mild steel is used in everything from ship construction to docks to large moorings. Marine corrosion is mitigated through measures such as coatings (paint or metal) or cathodic protection (Chandler, 1985); nonetheless it is still a pervasive problem. A significant amount of corrosion is initiated and/or exacerbated by the presence of microorganisms, a process referred to as microbiologically influenced corrosion (MIC). A number of different groups of microbes carrying out metabolisms such as sulfate reduction, methanogenesis, and Fe-reduction have been implicated in MIC, and it is known that these processes can enhance dissolution of steel and produce cathodic conditions that cause surface pitting which leads to more rapid corrosion (Little and Lee, 2007). A particularly aggressive form of corrosion, accelerated low water corrosion (ALWC) is commonly associated with microorganisms (Little and Lee, 2007; Dang and Lovell, 2016).

While the physiological attributes of different members of the MIC community are generally understood, less is known about the ecology of the process. For example, the make-up of naturally occurring MIC communities is still relatively unknown. Even less is understood about how MIC communities vary through space and time, and if there are temporal, spatial, or geographical patterns in the membership of marine corrosion communities. Thus, unraveling the complex relationships between these microbes, the steel surfaces they inhabit, the minerals they produce, and how they affect the corrosion process are all important areas of study.

One group that is worthy of consideration in this context, is the lithoautotrophic Fe-oxidizing bacteria (FeOB) that can use the ferrous iron (Fe(II)) released from the steel surface as an electron donor for growth (e.g., McBeth et al., 2011). Despite the fact that Fe(II) is their primary energy source and they are frequently identified in corrosion biofilms, the role of this group in MIC is still not clear. Recent studies have shown FeOB are able to colonize and grow either on steel coupons introduced into the environment (Dang et al., 2011; McBeth et al., 2011; Jin et al., 2015), or are present on corroded steel associated with ALWC structures (Marty et al., 2014). Laboratory studies have shown FeOB enhance release of iron from mild steel coupon surfaces, and that in concert with Fe-reducing bacteria they promote roughening of the steel surface (Lee et al., 2013). However, there is no evidence that FeOB are responsible for pitting or accelerated corrosion (Lee et al., 2013).

The goal of this study was to examine the development and succession of marine corrosion communities, and explore the role of iron-oxidizing bacteria (FeOB) in these systems. We hypothesized that sediment-derived marine FeOB would colonize and become numerically abundant on steel surfaces incubated in coastal marine environments. To test this, we incubated steel samples at two locations and sampled them at regular intervals over a 40-day period: in a tidal salt marsh stream and in a nearshore marine environment. Steel biofilm microbial community composition and succession was examined using high-throughput amplicon sequencing, single cell genomics, qPCR, and image analyses.

## Materials and Methods

### Sample Preparation, Deployment, and Sampling

Cold-finish 1018 mild carbon steel coupons (13 × 15 × 3 mm) were prepared as described in McBeth et al. (2011). Each coupon was contained in an individual subsampler, (see Supplemental Material Figure S2 in McBeth et al., 2011) that was composed of a cut off 15 ml conical tube with 9 μm mesh over the bottom and a black rubber stopper in the top. The subsamplers were contained in opaque PVC pipe holders with 1000 μm mesh bottom and opaque PVC caps. Samplers were sterilized by autoclaving and UV light illumination prior to addition of steel coupons and deployment at field sites.

The samplers were incubated at two locations. The GSB site was located in a tidal salt marsh on Great Salt Bay, Newcastle, ME (44° 2′29.73′′N; 69°31′55.78′′W). The GSB site study took place from 29 May to 9 July 2010 (sampled at 3, 6, 10, 14, 24, and 41 days). The second site location, designated the BBH site, was located just below the intertidal zone at the Maine Department of Marine Resources dock in West Boothbay Harbor, ME (43°50′39.50′′N; 69°38′27.29′′W) and took place from 16 August to 28 September 2011 (sampled at 3, 6, 9, 13, 17, 24, 31, 37, and 43 days). Tropical storm Irene moved through the region on 28 August 2011; however, the samplers remained in their original position and were undamaged. Samples for single cell genomics analyses were also deployed at the BBH site on 3 August 2011 and 16 August 2011 and were collected on 25 August 2011 (after 9- and 22-day incubations, respectively).

At each sampling timepoint, three steel coupons were collected and frozen for molecular analyses and a parallel coupon was collected and processed for imaging. Sediment samples immediately adjacent to the samplers were taken for comparative analysis. Corrosion products from each parallel coupon were examined using light microscopy to check for the presence of mineral biosignatures indicative of FeOB growth (Emerson et al., 2010), and samples from these coupons were also fixed for scanning electron microscopy analyses [1 ml + 0.1 ml 50% glutaraldehyde (Electron Microscopy Sciences)]. At each sampling, the site pH was measured with a portable pH meter, salinity was determined with a refractometer, and the water, air temperature, and water depth were recorded. The average values and standard error for each of these parameters were calculated over the course of each experiment.

For DNA analysis, frozen coupons in MoBio Powerwater kit tubes were thawed and subjected to the Powerwater kit (MoBio) DNA extraction protocol and eluted with 100 μl of PW6 buffer (10 mM tris buffer, pH 8). The extracts were assessed for quality and quantified with a nanodrop spectrophotometer (Thermo Scientific) and Qubit fluorometer dsDNA HS kit (Invitrogen). DNA samples were stored at -80°C until further analysis.

### Imaging

Scanning electron microscopy (SEM) samples were placed on filters and subjected to an ethanol dehydration series, mounted on aluminum stubs with carbon tape, critical point dried, and sputter coated with carbon. They were examined with a Zeiss Supra25 field emission SEM at a 10 kV accelerating voltage and 3 mm working distance. Samples of fresh biofilm from each series time point were examined using phase contrast light microscopy on an Olympus BX60 microscope with QICAM FAST CCD camera (QImaging, Surrey, BC, Canada).

### SSU rRNA Gene Community Analysis

High-throughput amplicon sequencing of the SSU rRNA gene was used to assess community composition and diversity of both steel coupon biofilms and sediments. DNA extracts from selected time points were shipped to Research and Testing Laboratory (Lubbock, TX, USA) and analyzed using 454 tagged pyrosequencing. Samples were amplified using the primers 530F (5′-GTGCCAGCMGCNGCGG-3′) and 1100R (5′-GGGTTNCGNTCGTTG-3′) which target the V4 variable region of the SSU rRNA gene in Bacteria, as detailed in Dowd et al. (2008). The samples were sequenced using 454 Titanium chemistry (Roche). The sequencing data were analyzed using mothur v.1.33.3 and the mothur 454 standard operating procedure^1^ (Schloss et al., 2009). Briefly, sequence barcodes and primers were removed, sequences were denoised with the mothur translation of the PyroNoise algorithm and trimmed to remove sequences if they contained ambiguous bases, homopolymers greater than 8, or less than 150 total bases. The remaining sequences were aligned to the SILVA bacterial database (alignment region start position 13858; Pruesse et al., 2007) and screened to remove sequences that were shorter than 250 bases without gaps. Remaining sequences were preclustered for sequences differing by a single base, and Uchime was used to detect chimeras (Edgar et al., 2011) measured against the Silva gold database. Chimeras were removed from the dataset. The resulting dataset was classified using the ribosomal database (RDP) classifier with a cutoff of 60% (Wang et al., 2007). The classifier database was modified with additional sequences and phylogenetic classification detail for the known FeOB (McBeth et al., 2013). The datasets from the 16 analyzed samples were normalized by random subsampling to include an equal number of reads (1173 each). The OTUs were clustered at a 97% similarity cutoff and classified. We calculated the Good’s coverage estimate, number of OTUs, Chao1 richness estimate, and inverse Simpson diversity estimate for each sample. Yue and Clayton Theta distance matrix values were calculated between the sample communities (OTUs at 3% distance) and used to construct non-metric multidimensional scaling (NMDS) ordination plots for the samples from each study area.

### Quantitative PCR

We conducted quantitative PCR (qPCR) analyses to assess the abundance of Zetaproteobacteria, sulfate-reducing bacteria (SRB), and Bacteria + Archaea in the DNA extracts from our samples. The primer sequences are summarized in **Table 1**; briefly, Zetaproteobacteria SSU rRNA gene copy numbers were quantified using primers designed by Kato et al. (2009), Bacteria + Archaea SSU rRNA gene copy numbers were obtained using primers designed by Takai and Horikoshi (2000), and SRB numbers were estimated using primers for the *DsrA* gene (Ben-Dov et al., 2007). The qPCR master mix contained Maxima SYBR Green qPCR Master Mix (Fermentas), primers at 0.3 μM, and 1 μl of template per reaction. Each reaction was prepared in triplicate and each plate also contained a standard dilution series of an amplified and quantified target gene, no template controls, and negative and positive control samples. For the Bacteria + Archaea and Zetaproteobacteria assays: standard series were prepared using amplified and quantified *Mariprofundus* sp strain GSB2 SSU rRNA gene (27F to 1492R; HQ206653); negative controls were prepared using amplified *Desulfovibrio salexigens* (DSMZ 2638) *DsrA* gene and the SSU rRNA gene of a *Bacillus* isolate (unpublished strain SV-1; 100% match over 878 bases for *Bacillus galliciensis* strain MER_190, KT719772), respectively, and the positive control was prepared from amplified and quantified SSU rRNA gene sample from an estuarine iron mat collected in a previous study (Sample ID Site 1 (b, 2011) in McBeth et al. (2013)). For the *DsrA* gene assay, the standard series and positive controls were prepared from amplified *Desulfovibrio salexigens DsrA* gene, and the negative control was prepared using the estuarine iron mat amplified SSU rRNA gene detailed above. Analyses were performed on an iq5 Thermal cycler (Biorad) and a C1000 Thermal cycler with CFX96 RT PCR Detection System (Biorad) using the recommended protocol for the Maxima SYBR Green qPCR master mix. Copy numbers were normalized to DNA template concentration (ng of DNA in extract).

**Table 1:** qPCR primers.

Target	Primers	5′-3′	Annealing/extension temperatures	Reference
Bacteria + Archaea (SSU rRNA gene)	Uni340F	CCT ACG GGR BGC ASC AG	55.8	Takai and Horikoshi, 2000
	Uni806R	GGA CTA CNN GGG TAT CTA AT		Takai and Horikoshi, 2000
Zetaproteobacteria (SSU rRNA gene)	Zeta672F	CGG AAT TCC GTG TGT AGC AGT	58	Kato et al., 2009
	Zeta837R	GCC ACW GYA GGG GTC GAT ACC		Kato et al., 2009
Sulfate-reducing bacteria (SRB) (*DsrA* gene)	RH1-dsr-F	GCC GTT ACT GTG ACC AGC C	55	Ben-Dov et al., 2007
	RH3-dsr-R	GGT GGA GCC GTG CAT GTT		Ben-Dov et al., 2007
				

### Single Cell Genomics

We single-cell sorted samples from the BBH site and conducted single-cell genomics analysis of the steel biofilms. The two sample points (9 and 22 days) were selected based on expected peak abundances of FeOB populations determined from our qPCR data observations from the GSB site time series. Each steel sample was placed in a 15 ml conical tube, 2 ml of filter sterilized seawater was added, the sample was gently agitated to dislodge flocculent microbial corrosion biofilm material from the coupon, and the biofilm material was transferred to a 2 ml centrifuge tube. The material was mixed five times with a 16G needle and syringe to break up particulates and microbes from the mineral matrix, and the material was gently homogenized using a vortex mixer. The samples were centrifuged briefly to remove large particles and diluted (1:10) in filtered seawater. The diluted samples were immediately sorted at the Bigelow Laboratory Single Cell Genomics Center (SCGC) for particles falling in the HNA region of the dot plot (medium sized particles of high fluorescence, likely to be bacteria). Samples were amplified using multiple displacement amplification (MDA), and screened for the presence of bacteria using the primers 27F (3′AGR GTT YGA TYM TGG CTC AG-5′) and 907R (3′-CCG TCA ATT CMT TTR AGT TT-5′) (Lane et al., 1985, 1991; Page et al., 2004). The amplicons were sequenced at the Beckman Coulter Genomics Facility^2^ and the contigs were assembled in Sequencher (Gene Codes, Ann Arbor, MI, USA) and manually curated to correct mislabeled bases and remove sequences of poor quality. Sequences were aligned and classified using mothur as described for high-throughput amplicon sequencing analyses. Neighbor-joining trees were constructed for SSU rRNA gene data from Epsilonproteobacteria and Zetaproteobacteria single amplified genomes (SAGs) using Jukes–Cantor correction in ARB (v. 5.3, database 108). Percentages represent the bootstrap values at each node for 1000 replicate trees; percentages less than 50% are not shown. Cultured and uncultured nearest matches are presented in the trees to identify possible physiology/metabolism of SAGs.

The high-throughput amplicon sequencing libraries and SAG sequence libraries were deposited at the European Nucleotide Archive (ENA) under the study accession numbers PRJEB12866 (sample accessions numbers ERS1068403 to ERS1068426) and PRJEB12879 (secondary accession number ERP014404; sample accession numbers 1069314 to ERS1069573), respectively.

## Results

### Site Conditions

At the GSB saltmarsh site, the sampler was placed on soft sediments typical of a tidal saltmarsh. At the low water point, the sampler was above the water surface. The sampler was exposed to diurnal variations in temperature, salinity, and saturation (detailed in **Table 2**). We observed a salt wedge in the stream when the tide was high; higher salinity water inundating the salt marsh at high tide and freshwater flowed out in the stream at low tide. As the study progressed, the sample holders accumulated corrosion products and sediment particulates which caused them to retain water even during low tide.

**Table 2:** Site conditions over the course of the studies.

Site	Parameter	Mean minimum	Mean maximum	Average	Maximum	Minimum	Total	Reference
GSB	Precipitation						352 mm	Newcastle, ME weather station (NOAA, 2015)

GSB	Air temperature	12.4°C	23.3°C		33°C	-2°C		Newcastle, ME weather station (NOAA, 2015)

GSB	Measured air temperature	18°C	27°C	22.2 ± 0.6°C				This study

BBH	Measured air temperature			19 ± 1°C	23°C	14°C		This study

GSB	Stream temperature			17 ± 0.8°C				This study

BBH	Sea surface temperature (±0.2°C)	16.3°C	18.5°C		21.4°C	13.4°C		(Maine DMR, 2015)

BBH	Measured water temperature			19 ± 1°C	21°C	13°C		This study

GSB	Stream pH			6.6 ± 0.1				This study

BBH	pH			8				This study

GSB	Stream salinity			6 ± 2‰	21‰ (high tide)	0‰ (low tide)		This study

BBH	Measured salinity			30.2 ± 0.5‰	32‰	27‰		This study

GSB	Stream depth				37 cm	1.5 cm		This study

GSB	Salinity at end of saltmarsh			26 ± 1‰				This study

At the BBH site, the samples were placed in a protected boat harbor on soft bottom, approximately 0.3 m below the extreme low water mark for the tides during the course of this study. The maximal tidal range during the study was on the order of 3.5 m. These samplers were exposed to only minor changes in salinity and temperature over the course of the study (**Table 2**).

### Macroscopic Corrosion Evidence and Imaging Results

**FIGURE 1:**
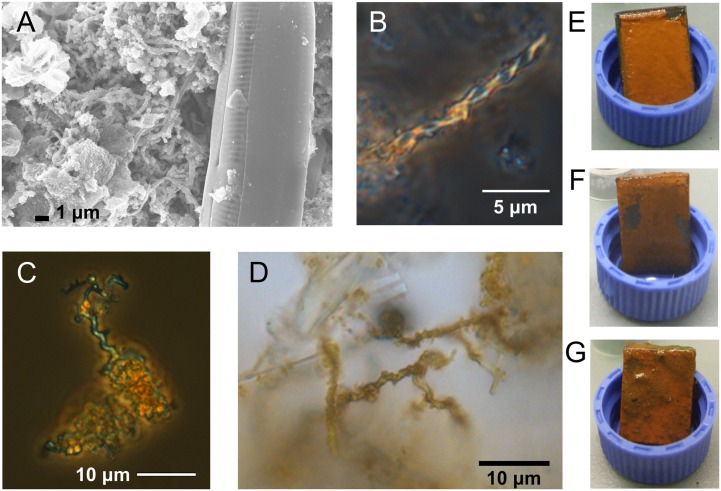
**Microscopy images of stalk structures characteristic of FeOB from field incubations and macroscopic images of corrosion biofilms. (A)** SEM image from GSB site day 6, **(B)** light microscopy image from GSB site day 41, **(C)** light microscopy image from BBH site day 6, and **(D)** light microscopy image from BBH site day 13. Macroscopic images of BBH biofilms at: **(E)** day 9, **(F)** day 13, and **(G)** day 43.

The steel coupons developed orange flocculent coatings within the 1st week of each incubation; by the end of each study the coatings were still predominantly orange but also included patches of black or greenish precipitates (**Figures 1E–G**). Stalk structures indicative of FeOB were observed in microscopy images of the steel corrosion products at timepoints throughout the incubations (**Figures 1A–D**).

### High-throughput Amplicon Sequencing Results

**FIGURE 2:**
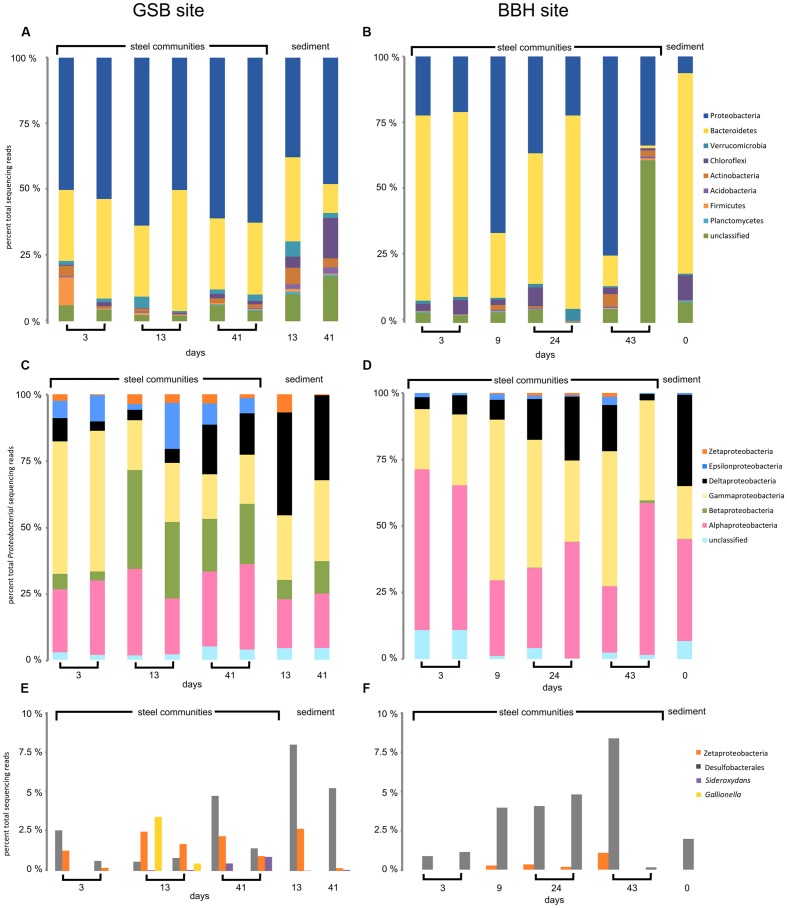
**High-throughput amplicon sequencing data for GSB saltmarsh and BBH harbor sites. (A,B)** major phyla, **(C,D)** classes of Proteobacteria, and **(E,F)** sequences of FeOB relatives and Desulfobacterales.

**FIGURE 3:**
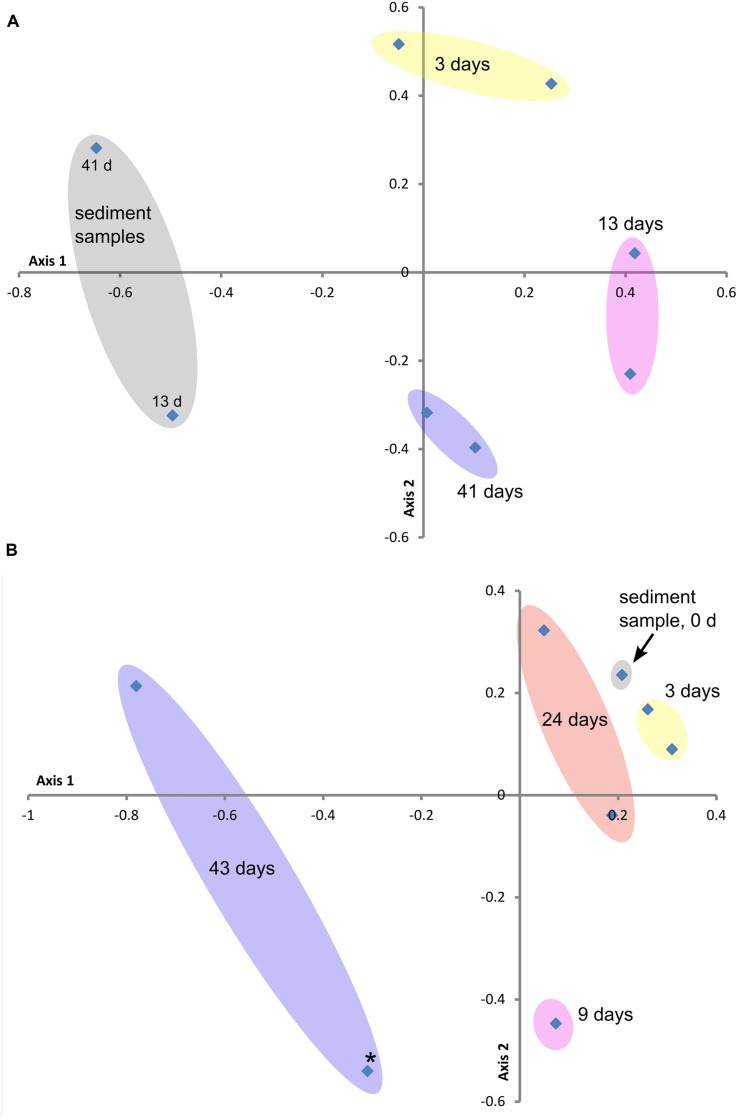
**Beta diversity ordination plots of sample communities from the two study sites: **(A)** GSB site and **(B)** BBH site.** These non-metric multidimensional scaling plots (NMDS) are based on the Yue and Clayton Theta measure of dissimilarity values (distance matrix calculated at the 3% level), which assesses structural differences between the sample communities. Stress and *R*^2^ values for these ordination plots were **(A)** 0.20 and 0.76 and **(B)** 0.09 and 0.97, respectively. Colored ovals encompass replicate samples where applicable. Point marked with star (^∗^) in plot **(B)** represents outlier sample from **Table 3**.

Amplicon sequencing was used to understand the community dynamics of populations colonizing steel coupons over time at the brackish site and full strength seawater site. We obtained between 1,173 and 44,683 reads for each of the coupon and sediment samples. The results of replicate coupon and sediment analyses are shown in **Figures 2** and **3**. Two samples from the BBH site (one of the two 9-day coupons and the 43-day sediment sample) failed in PCR amplification, and so were eliminated from the dataset. Overall there were consistent patterns of taxonomic distributions between replicates, with one notable exception being the 43-day coupon samples from the BBH site, where unclassified reads dominated the total number of bacterial reads from one of the two replicate samples (**Figure 2B**) This sample was an outlier compared to the patterns in richness and diversity seen in the other 43-day coupon from this site, which was more consistent with the other BBH samples from the time-course.

At the phylum level, the corrosion biofilm and sediment communities in both series were dominated by Proteobacteria and Bacteriodetes (**Figures 2A,B**). Within the Proteobacteria, Zetaproteobacteria were present on the GSB site coupons throughout the experiment, as well as in sediments adjacent to the steel samples (**Figure 2E**). The presence of Zetaproteobacteria in the saltmarsh sediments was consistent with small but visible iron mats we observed adjacent to the incubation chamber at the GSB site. At the BBH site, sequences from the Zetaproteobacteria were present from 9 days onward, but were not observed in the sediments (**Figures 2D,F**).

A substantial difference between the GSB and BBH sites was the presence and relative abundance of the families Comamonadaceae and Gallionellaceae (both Betaproteobacteria) at the brackish GSB site, but their absence at the BBH site. At GSB, the most abundance genera within these families were related to *Hydrogenophaga, Rhodoferax, Nitrotoga*, and the known FeOB *Gallionella* and *Sideroxydans.*

Another notable difference between the sediment and steel biofilm community samples was the abundance of Epsilonproteobacteria. In the corrosion communities, Epsilon proteobacteria accounted for 2–17% (GSB site) and 0.1–2% (BBH site) of the total Proteobacteria reads, while in the sediment samples at both sites they were barely detectable, accounting for<1% of total Proteobacteria reads (**Figures 2C,D**). At the GSB site, the predominant genera of Epsilonproteobacteria were related to sulfur-oxidizers *Sulfurimonas*, *Sulfuricurvum*, and *Sulfurovum*. Relatives of a sulfide-oxidizing *Arcobacter* species also represented a large portion of the Epsilonproteobacteria in the early GSB biofilm samples, but their abundance decreased over the course of the incubation. In the BBH site steel biofilms relatives of *Sulfurimonas, Sulfurovum, and Arcobacter* were present.

The Deltaproteobacteria on the steel biofilms increased through time in each series and reads from this class were high in the sediment samples, as expected (**Figures 2C,D**). At the GSB site, many of these Deltaproteobacteria reads were Desulfobacterales (**Figure 2E**), Desulfuromonadales, and Myxococcales, and included relatives of known sulfate- and iron-reducing bacteria. At the BBH site, many of the reads also classified as Desulfobacterales (**Figure 2F**) and Myxococcales; however, fewer reads classified as Desulfuromonadales. These orders included reads that classified as relatives of the sulfate- and iron-reducing bacteria but in contrast to the GSB data, we did not observe reads classifying as *Geobacter* or *Geopsychrobacter* in the BBH site data.

The Alphaproteobacteria reads at both sites were dominated by the Rhodobacteriaceae; generally speaking, Alphaproteobacteria were richer in the GSB site samples in comparison with the BBH site samples. Gammaproteobacteria at the GSB site included relatives of methanotrophs, methane-oxidizers, and sulfur oxidizers, and at the BBH site we identified relatives of several known sulfur oxidizers. Reads classified as Shewanellaceae, potential iron-reducers, were identified in both data sets.

The amplicon results revealed members of the Bacteroidetes were remarkably abundant at the BBH site. In the BBH sediment sample, they accounted for nearly 80% of relative abundance of all the bacterial reads. These percentages are much higher than have been seen in other coastal sediments (Wang et al., 2012). Consistent with the high sediment abundance of Bacteroidetes at the BBH site, the day 3 coupons at the BBH site had approximately 70% Bacteroidetes. Over time their relative abundance was variable but showed a general decline with biofilm age (**Figure 2B**). At the GSB site, the presence of this phylum was relatively constant and accounted for between 10 and 25% of the community. At both sites, the Bacteroidetes were dominated by the class Flavobacteria, which contains a diverse group of aerobic heterotrophs.

Alpha diversity results from the time series experiments are summarized in **Table 3**. The Good’s coverage estimate indicated that the amplicon sequencing analyses captured between 63 and 98% of the sample community composition. Overall, the GSB site samples were richer and more diverse than the BBH samples. The sediment samples at the GSB site had particularly high diversity estimates, exceeding the other samples by nearly an order of magnitude. Zetaproteobacteria were richer at GSB (21 OTUs at 97% similarity) than at BBH (1 OTU).

**Table 3:** Alpha diversity results for high-throughput amplicon sequenced samples from GSB site and BBH site.

Site	Sample (days from start of time series, replicate)	Good’s coverage estimate	Number of observed OTUs at 97%	Chao1 richness estimate	Inverse Simpson diversity estimate	Sample ID and ENA reference
GSB site	Steel (3, a)	0.86	351	566	115	GSB-FTS1-T1a.sff, ERS1068403
	Steel (3, b)	0.81	360	761	61	GSB-FTS1-T1b.sff, ERS1068404
	Steel (13, a)	0.79	384	933	62	GSB-FTS1-T4a.sff, ERS1068405
	Steel (13, b)	0.84	306	724	33	GSB-FTS1-T4b.sff, ERS1068406
	Steel (41, a)	0.79	398	908	70	GSB-FTS1-T6a.sff, ERS1068407
	Steel (41, b)	0.78	412	924	67	GSB-FTS1-T6b.sff, ERS1068408
	Sediment (13)	0.67	544	1592	111	GSB-FTS1-T4sed.sff, ERS1068409
	Sediment (41)	0.63	664	1436	467	GSB-FTS1-T6sed.sff, ERS1068410
BBH site	Steel (3, a)	0.94	156	247	11	BBH-FTS3-T1b.sff, ERS1068414
	Steel (3, b)	0.95	153	224	14	BBH-FTS3-T1c.sff, ERS1068415
	Steel (9)	0.88	288	490	61	BBH-FTS3-T3a.sff, ERS1068416
	Steel (24, a)	0.86	300	617	25	BBH-FTS3-T6a.sff, ERS1068418

*All samples subsampled to 1173 sequences, and analyses were calculated for OTUs at 97% similarity*.

**Figures 3A,B** contain the Beta diversity results. At both field sites we observed a trend in the community structure changes over the course of the experiments. As in the sequencing and qPCR data, we observed more variability in the replicate data and over the course of the experiment at the BBH study than at GSB. The initial timepoint samples from BBH clustered close to the sediment sample from that site.

### qPCR Results

**FIGURE 4:**
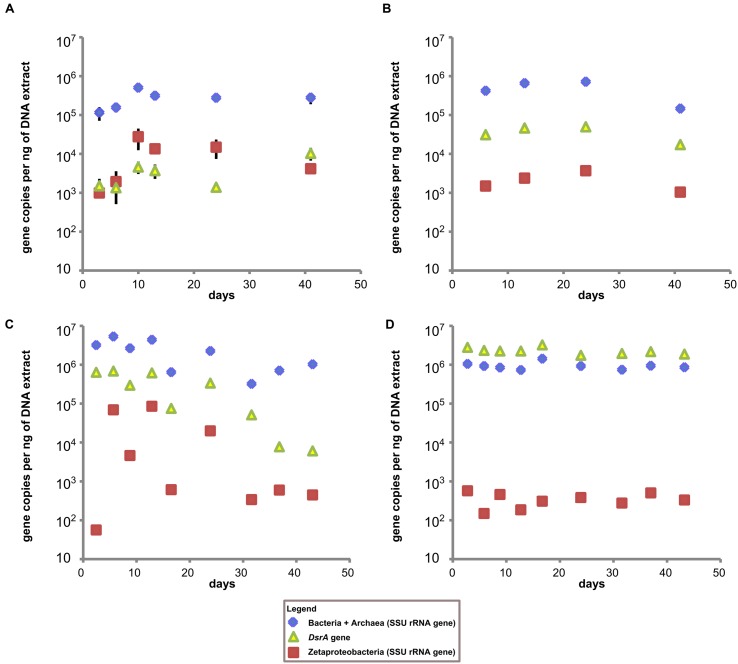
**Time series qPCR results. (A)** Results from GSB site duplicate steel coupon extracts, **(B)** results from single stream sediment samples taken from sediment adjacent to the GSB site sampler, **(C)** results from BBH site triplicate steel coupon extracts, and **(D)** results from single sediment samples taken adjacent to the BBH site sampler. All data is gene copies per ng of DNA in extract. Error bars represent standard error of replicate samples where applicable; if not visible they fall within the boundaries of the symbol.

The low total DNA recoveries from the coupon surfaces (1–3 ng/ul for most GSB site samples and <1 ng/ul for most BBH site samples) made qPCR analyses challenging, and resulted in less than optimal replication between coupon samples. Nonetheless, for the coupons incubated at the GSB site, the Bacteria + Archaea SSU rRNA gene copies remained relatively constant over the course of the incubation at 10^5^–10^6^ gene copies per ng of DNA (**Figures 4A,B**) for both the coupons and the sediments. At the BBH site the Bacteria + Archaea SSU rRNA gene copy numbers on coupons spanned a broader range with 10^5^–10^7^ gene copies per ng of DNA, but were consistently on the order of 10^6^ gene copies per ng of DNA in the sediments (**Figures 4C,D**).

Estimates of the abundance of Zetaproteobacteria on steel coupons at the GSB site based on qPCR indicated they were already detectable at 3 days, increased up to 10 days, and then declined gradually over the next 30 days. The copies of the *DsrA* gene (indicative of SRB) at the GSB site increased gradually over the course of the experiment from about 10^3^ up to 10^4^ gene copies per ng of DNA. The qPCR data for the GSB site sediment was relatively constant, with Zetaproteobacteria about 1 to 2 orders of magnitude less abundant than SRB, and about 1 to 2 orders of magnitude less abundant than their maximal abundance measured on the coupons (at 10 days).

In contrast, at the BBH site the *DsrA* gene copies started out at about 10^6^ gene copies per ng of DNA and decreased gradually over time, with a final concentration around 10^4^ gene copies per ng of DNA. Here the Zetaproteobacteria abundance was essentially at or below the assay detection limit at 3 days, but then showed a large increase in abundance by days 6 and 13, followed by fluctuations and a general decline over time. In the BBH site sediments, Zetaproteobacteria were at concentrations at or below the detection limit of the assay throughout the study, and *DsrA* gene copies were on the order of 10^6^ gene copies per ng of DNA. Note that the *DsrA* primers used in our study are known to overestimate the abundance of *DsrA* genes present which is a likely explanation for why these gene copies outnumber the total Bacteria + Archaea gene copies in **Figure 4D** (Ben-Dov et al., 2007).

### SAG Results

**FIGURE 5:**
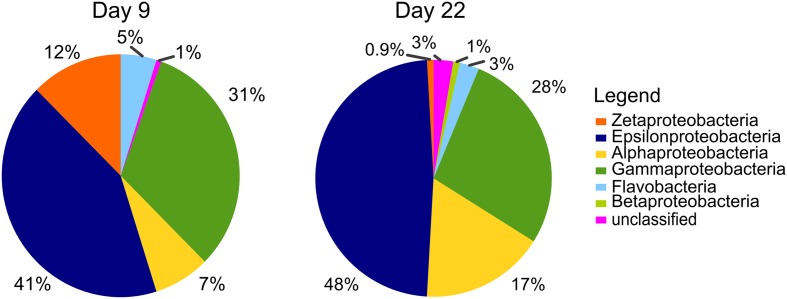
**Single amplified genome (SAG) results from BBH site, results from bacterial SSU rRNA gene screen of plates sorted from day 9 (148 SAGs) and day 22 (118 SAGs).** Pie charts show percent of each class of Proteobacteria observed in each sample.

**FIGURE 6:**
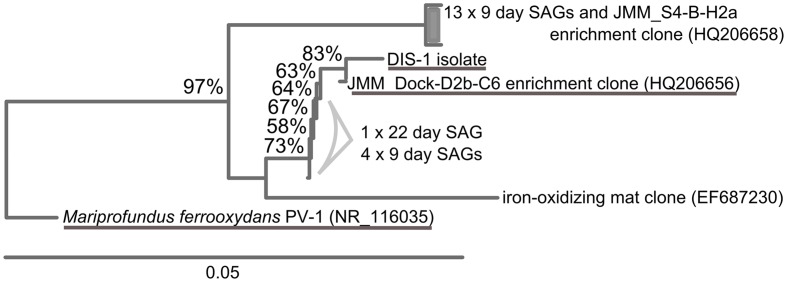
**Neighbor-joining tree showing SAGs classified as Zetaproteobacteria identified in the BBH site single cell sorts.** Stalk-forming FeOB Zetaproteobacteria related to the SAGs are underlined.

Single cell sorts from the BBH site were successful and resulted in identifiable sequences from 148 cells (9-day sample) and 112 cells (22-day sample). Pie charts illustrating the proportions of taxa present in the SAG results are summarized in **Figure 5**. Zetaproteobacteria comprised 12% of the bacterial SAG library from the 9-day coupon sample, while at 22 days, only 0.9% of the SAG library were Zetaproteobacteria, indicating the proportion of Zetaproteobacteria in the biofilm communities decreased with time. Interestingly, the Zetaproteobacteria observed in both samples represented only two clonal sequences (**Figure 6**), both previously identified at this site and in other coastal enrichment studies (Genbank accession numbers HQ206658 and HQ206656; McBeth et al., 2011). No other relatives of known FeOB were identified in the SAG analyses.

**FIGURE 7:**
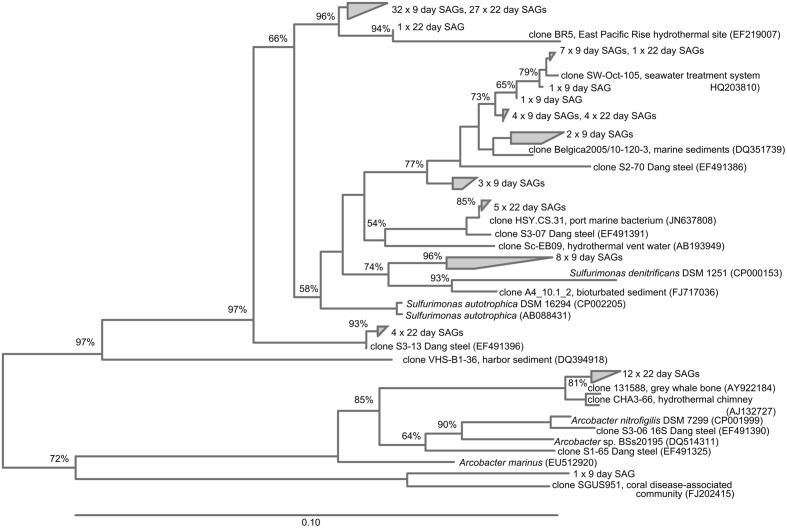
**Neighbor-joining tree showing SAGs classified as Epsilonproteobacteria identified in the BBH site single cell sorts**.

Epsilonproteobacteria were numerically important corrosion community members in the SAG analyses (41 and 48% of the SAG libraries at 9 and 22 days, respectively; **Figure 5**). All of these SAGs grouped with relatives of the known sulfur-oxidizing genera *Sulfurimonas* and *Arcobacter*; in addition the day 9 SAG library had one *Sulfurovum* relative. Again, several clonal populations were observed in the SAG libraries (**Figure 7**). The Gammaproteobacteria (28–31% of the bacterial SAG libraries) were diverse at day 9 timepoint, but by 22 days the Gammaproteobacteria were reduced to relatives of *Thiomicrospira*. Alphaproteobacteria (7–17%) and Flavobacteria (3–5%) were also present. In addition to these recognized phylogenetic groups identified in the SAG libraries, 2 SAGs were only 90–92% matches to any know Genbank sequence.

## Discussion

The work presented here shows that mild steel corrosion communities are dynamic over time and there are successional patterns in the microbial communities. In both the brackish and seawater incubations, members of the Zetaproteobacteria that are presumptive FeOB were enriched on the steel surface, confirming earlier studies that FeOB are an important part of the MIC community (Dang et al., 2011; McBeth et al., 2011). At GSB, incubations were done in a locale where there was visible Fe-oxidation, and presumably Fe-reduction, occurring at the sediment interface in proximity to the steel coupons. In this case the Zetaproteobacteria were detectable in the sediments, but were enriched by 1 to 2 orders of magnitude on the coupons. The incubation site at the fully marine BBH site was on soft bottom sediment with no visible evidence for Fe-cycling (i.e., presence of iron mats or orange sediment), and qPCR assays did not detect Zetaproteobacteria in the surface sediments. Nonetheless, the same pattern of rapid colonization of the steel surface by FeOB occurred, and based on qPCR data, the abundance of Zetaproteobacteria on the steel surface reached a similar order of magnitude as for the brackish site at GSB. In both cases, it appeared the Zetaproteobacteria population reached an apex after 10–12 days and then declined. These results show that FeOB are early colonizers of steel surfaces, and are consistent with the findings of Dang et al. (2011). However, as the community develops, their relative abundance decreases, suggesting that the steel surface niche is changing to become a more mature biofilm with anoxic zones that support the growth of strict anaerobes like SRB. This is consistent with previously models for mature steel corrosion communities (Hamilton, 1985; Little and Lee, 2007).

This study is unique in looking at both brackish and marine environments with initiation of corrosion under fully aerobic conditions, nor are we aware of studies, aside from the work of Dang et al. (2011), that have investigated the *in situ* population dynamics of MIC communities. Over the 40-day incubation course of these experiments, the total diversity of the communities did not increase significantly; however, the population structure did change consistently through time (**Figure 3**). These dynamics indicate that the surface niche has not stabilized and that continued physicochemical changes may select for different populations, while at the same time ecological interactions between populations are also continuing to shape and alter the community. A longer time course would be necessary to determine if the MIC community reaches a stable composition. Deltaproteobacteria were expected to be part of the community, since this class includes a known sulfate- and Fe-reducing genera and both of these physiological groups have been previously observed in association with marine MIC communities (e.g., Dang et al., 2011; Enning et al., 2012). The relative abundance of Deltaproteobacteria, as assessed by amplicon sequencing, increased with time at both sites. The dynamics of SRB as specifically assessed by qPCR differed between GSB and BBH. At GSB, copy numbers of *DsrA* were low at 15 days and then increased over time, this is consistent with formation of a more mature biofilm with anoxic niches that could support sulfate-reduction. At BBH, *DsrA* copies were relatively abundant initially, on a par with the sediments, and then decreased over time, while sediment numbers stayed quite constant. We do not have a good explanation for this pattern, and it is inconsistent with the relative increase in reads of Deltaproteobacteria in the amplicon analysis. One possibility is that Fe-reducing Deltaproteobacteria may have been a larger part of the community than SRB at the early timepoints, however, this is not reflected in the classification of the sequencing results. It has also recently been shown that some Alphaproteobacteria belonging to the *Roseobacter* clade contain *DsrA* genes and it is possible they may have also been targeted by the qPCR primers (Lenk et al., 2012).

The relatively high percentages (typically ranging from 25 to 75%) of Bacteroidetes in comparison with other sites (Wang et al., 2012) from sediment and coupon surface amplicon libraries was notable (**Figures 2** and **3**). The presence of Bacteroidetes on the BBH sampler coupons through most of the time course was especially pronounced. They accounted for nearly 70% of reads during the middle of the time-course, although they declined significantly by 43 days (**Figure 2**). A more detailed assessment of the SSU rRNA amplicon reads for this group indicated the closest known relatives were members of the Flavobacteriales that includes aerobic and facultative heterotrophs that are often saprophytic (Krieg et al., 2010). The work of Dang et al. (2011) also showed abundant Flavobacteriales on the surface of steel coupons incubated in coastal waters in China. The specific role of this group in the MIC community is difficult to ascertain, although it is possible they are taking advantage of primary production by chemolithotrophic Fe- and S-oxidizing bacteria. It is also known that members of the Flavobacteriales are efficient at colonizing surfaces and growing as biofilms (Doghri et al., 2015); thus, they may preferentially colonize substrates that are newly introduced into their environment. Another possible explanation for the high abundance of Bacteroidetes at the BBH site is that they are often excellent organic matter decomposers, and the environment was rich in particulate organic matter. More specifically, there was a significant amount of seaweed in the vicinity of the sampler, although it was not in direct contact with the samples. This seaweed was starting to undergo senescence and degradation in late summer, which would likely promote the growth of saprophytic microbes (Thomas, 2015).

The high number of Epsilonproteobacteria identified in the SAG libraries at both time points was interesting. These represent absolute cell numbers in the sorted sample, which included cells from the loosely adhering portion of the biofilm. The Epsilonproteobacteria were consistently present in the amplicon libraries, but at a lower relative abundance. Members of the Epsilonproteobacteria are not commonly reported as being important members of the MIC community; however, Dang et al. (2011), also noted their relative abundance in steel colonization, and suggested they might be involved in S-oxidation, perhaps coupling their metabolism with the reduced S-products of sulfate reducers. Our findings are consistent with that report, as the identified genera included *Sulfurimonas* and *Arcobacter*, known S-oxidizers (Campbell et al., 2006). However, it cannot be ruled out that some of these organisms may also be carrying out H_2_-oxidation (e.g., Takai et al., 2006), since H_2_ production commonly occurs at steel surfaces (Little and Lee, 2007).

The presence and relative abundance of Zetaproteobacteria SAGs on the 9-day BBH samples in comparison with the samples taken at 22 days was consistent with the general decline in Zetaproteobacteria abundance observed from qPCR over the same timeline. It is worth noting we did not obtain any Deltaproteobacteria in the SAG libraries. Although it is possible this is bias of selecting a particular population of high nucleic acid cells via flow cytometry sort or bias against either lysis or amplication of this group in the SAG preparation, we believe a more likely explanation may have to do with the structure of the biofilm, and how it was sampled. The samples for SAG analysis were taken only from a comparatively loose surface layer of the biofilm, where more aerobic members of the community would be expected. For high-throughput amplicon sequencing and qPCR analyses the entire surface layer (consisting of the outer, loosely adherent layer, and an inner, more adherent layer) was used in order to obtain enough nucleic acid for the analyses. Thus it is possible the SAG libraries are selecting organisms with more aerobic metabolisms, which is consistent with the relative abundance of Zetaproteobacteria and Epsilonproteobacteria.

The population structure of FeOB within the biofilms showed important differences between brackish and fully marine conditions. At GSB, where the salinity fluctuated from between 0 and 21 ppt as a result of tidal influence, representatives of the freshwater FeOB, Gallionellaceae were found in addition to Zetaproteobacteria. This is consistent with an earlier study that looked at the distribution of FeOB along a salinity gradient and found overlap between these groups in brackish regions (McBeth et al., 2013). This also implies that freshwater FeOB are part of MIC communities in strictly freshwater habitats, and is consistent with their presence on steel corrosion products in the Great Lakes (Hicks, 2007), scale from reclaimed wastewater (Jin et al., 2015), and from an incubation experiment (Rajala et al., 2015). Interestingly, the richness of Zetaproteobacteria was far greater at GSB – 21 OTUs at 97% similarity – than it was in fully marine BBH where only a single OTU was observed. In fact, our analysis of Zetaproteobacteria SAGs from BBH indicated a very low diversity with only two clones accounting for the abundance of Zetaproteobacteria on the samples. One explanation for this is that a low overall abundance of Zetaproteobacteria in the coastal ocean (at least at this location) leads to recruitment of only a few strains that become established on the steel surface and grow to abundance. This observation is supported by the clonal Zetaproteobacteria sequences we were able to amplify in a previous coastal study of corrosion biofilms (McBeth et al., 2011). Interestingly, basalt coupons incubated in the abyssal plain of the deep ocean are also known to recruit Zetaproteobacteria that presumably gain energy from the reduced iron within the basalt. These communities are also of low diversity, suggesting a similar mechanism of recruitment (Henri et al., 2015), as predicted by Edwards et al. (2004).

## Conclusion

In this study, we have characterized the succession of mild steel corrosion communities over 40 days in both nearshore marine and estuarine salt marsh environments. This is the first time a detailed study of corrosion community succession has been conducted over a period of several weeks. We found that iron-oxidizing Zetaproteobacteria and Betaproteobacteria were able to rapidly colonize the steel surfaces over the first 10 days of the incubations; however, their numbers dropped after this initial colonization and as the corrosion community matured on the steel surfaces. Epsilonproteobacteria were also enriched on the steel samples over the course of these experiments, relative to background sediment samples. The iron-oxidizing Zetaproteobacteria were still able to achieve rapid colonization in the coastal marine environment where their ambient abundance was extremely low. This is further evidence of the amazing ability of these microbes to flourish on sources of reduced iron provided by transient niches as the opportunity arises – whether that niche consists of a steel structure or a fresh pillow basalt deposit.

## Author Contributions

This study was conceived by DE and designed by DE and JM. JM conducted the data acquisition, analysis, and interpretation, and manuscript preparation with assistance from DE. JM and DE wrote the manuscript, and have reviewed the final version for submission for publication. JM and DE agree to be accountable for all aspects of the work in ensuring that questions related to the accuracy or integrity of any part of the work are appropriately investigated and resolved.

## Conflict of Interest Statement

The authors declare that the research was conducted in the absence of any commercial or financial relationships that could be construed as a potential conflict of interest.

## Footnotes

1 http://www.mothur.org

2 http://www.beckmangenomics.com
